# Datasets describing optimization of the cutting regime in the turning of AISI 316L steel for biomedical purposes based on the NSGA-II and NSGA-III multi-criteria algorithms

**DOI:** 10.1016/j.dib.2023.109475

**Published:** 2023-08-09

**Authors:** Hiovanis Castillo Pantoja, Alexis Cordovés García, Roberto Pérez Rodríguez, Ricardo del Risco Alfonso, José Antonio Yarza Acuña, Ricardo Lorenzo Ávila Rondón

**Affiliations:** a University of Holguín, CAD/CAM Study Center, Holguín, Cuba; b UTE University, Quito, Ecuador; c University of Camaguey, CEEFREP Study Center, Camaguey, Cuba; d Autonomous University of Coahuila, Saltillo, Mexico

**Keywords:** AISI 316L, Optimization, NSGA-II, NSGA-III, Dry, MQL

## Abstract

There are several methods of analysis used in the metalworking industry for dry machining processes and with Minimum Quantity Lubrication (MQL). Evolutionary methods [1] have been used in the decision-making process in the machining process to select the optimal data and to analyze the behavior of variables such as cutting speed (V), feed rate (f) and cutting depth (a_p_). This work addresses the use of evolutionary algorithms of low dominance class II and III (NSGA-II and NSGA-III) to analyze from the multicriteria approach the initial wear of the cutting tool (VB), the energy consumption (E) and the machining time (t) in the turning process of the AISI 316L steel workpiece for biomedical purposes. As input variables to the algorithm with 54 records, there are: cutting speed (V: 200, 300, 400 m/min) and feed rate (f: 0.1, 0.15, 0.2 mm/rev). The experiment was developed for a dry (1) turning operation and with the use of MQL (-1). For the MQL lubrication regime, a TRI-COOL MD-1 lubricant was employed, a vegetable type used in ferrous and non-ferrous metal cutting operations. A BIDEMICS JX1 ceramic cutting tool was used.

Specifications Table

**Table:** 

Subject	Manufacturing Engineering; Mechanical Engineering
Specific subject area	Minimizing the initial progression of tool wear based on the optimal selection of parameters in the turning of AISI 316L steel workpiece for biomedical purposes with dry and MQL, and more efficient use of energy consumption
Type of data	Table
How the data were acquired	Data was collected on paper and later transferred to an Excel spreadsheet. The average hardness was obtained with a Wilson Rockwell durometer. A Kistler 9257B dynamometer was used to measure cutting forces; and a Mitutoyo SJ-201P roughness tester to measure surface roughness. Wear was measured on the relief surface of the cutting tool with the help of a Zeiss EVO MA25 scanning electron microscope. The measurements were carried out in a turning operation with the different cutting parameters mentioned above. As a machine tool, a HAAS ST10 CNC lathe was used with a maximum capacity of 356×406 mm, 11.2 kW of power and 6000 rpm in the spindle
Data format	Raw
Description of data collection	The experiment was developed for a dry (1) turning operation and with the use of MQL (-1). The cutting speed (V) in three levels (200, 300 and 400 m / min; 1, 0, -1) and the feed rate (f) in three levels (0.1, 0.15 and 0.2 mm / rev; 1, 0, -1) were taken as independent variables, three replications were made of each run (Table 1). For the MQL lubrication regime, the TRI-COOL MD-1 lubricant of vegetable type was used for cutting operations. In the output variables, the main cutting force (Fz), cutting tool wear (VB), and surface roughness (Ra) are measured
Data source location	University of Holguín, CAD/CAM Study Center, Holguín, Cuba
Data accessibility	Data identification number: doi:10.17632/vxwkhpc3z6.2 Direct URL to data: https://doi.org/10.17632/vxwkhpc3z6.2
Related research article	del Risco-Alfonso, R., Pérez-Rodríguez, R., Zambrano Robledo, P.d.C., Rivas Santana, M., and Quiza, R., Optimization of the Cutting Regime in the Turning of the AISI 316L Steel for Biomedical Purposes Based on the Initial Progression of Tool Wear. Metals, 11 (2021) 1698. https://doi.org/10.3390/met11111698

## 1 Value of the Data

• These data are important, since they facilitate in the technological process of machining AISI 316L steel for biomedical purposes, with MQL and dry, to predict the initial wear of the cutting tool through the multicriteria method with artificial intelligence. The algorithms used facilitate the determination of the hierarchy of the best alternative in a very short time.
• The data can be used in research related to decision-making in the AISI 316L machining process for biomedical purposes, evaluating the best results in terms of production quality and low costs, for workshops or companies in the metalworking sector.
• The mathematical optimization model used, with multicriteria analysis, quickly evaluates the best values of the dependent variables in the two environments (dry and MQL) analyzed.

## 2 Objective

The optimization of manufacturing processes by material removal is a very active area of study in the scientific literature. By applying optimization tools, the management of operations in machining workshops is improved. With the optimal data, it is possible to increase production, guaranteeing greater durability of the cutting tools. With the interpretation of the data from the research "Optimization of the Cutting Regime in the Turning of the AISI 316L Steel for Biomedical Purposes Based on the Initial Progression of Tool Wear" [1], it was performing a multicriteria analysis of the dry machining operation and MQL of 316L steel for biomedical purposes. By applying the NSAG-III algorithm as a new generation of the non-dominant evolutionary genetic algorithm, and with the input data of the cutting speed (V) and the feed rate (f), it is possible to establish the hierarchy of solution variants with the recommended parameters.

## 3 Data Description

The data set from the results (Table 1) of the experiment was used, when machining AISI 316 steel specimens for biomedical purposes with a hardness of 148 HRB on a CNC lathe. A BIDEMICS-type ceramic tool was used as a cutting tool. A HAAS ST10 CNC machining center was used as a machine tool.

**Table 1:** Data of the experimental values of the machining of AISI 316L steel for biomedical purposes. It includes 2 columns and shows the 54 measurements made to the specimens in the dry turning and MQL operation. As input variables we have cutting speed (V), feed rate (f) and lubrication regime (LR). As output variables we have wear of the cutting tool (VB), surface roughness (Ra) and cutting force (Fz).

	INPUT PARAMETERS			OUTPUT PARAMETERS				INPUT PARAMETERS			OUTPUT PARAMETERS		
Idx	V	f	LR	VB	Ra	Fz	Idx	V	f	LR	VB	Ra	Fz
1	300.00	0.20	1	0.077	0.30	248.17	28	300.00	0.20	1	0.076	0.41	240.30
2	200.00	0.10	-1	0.044	0.38	290.97	29	200.00	0.20	1	0.096	0.33	220.81
3	200.00	0.10	1	0.075	0.54	340.11	30	400.00	0.15	-1	0.055	0.48	290.72
4	400.00	0.15	1	0.060	0.55	310.98	31	300.00	0.15	1	0.074	0.49	288.98
5	300.00	0.20	-1	0.054	0.30	229.22	32	400.00	0.20	1	0.063	0.57	289.99
6	400.00	0.20	1	0.062	0.53	290.87	33	200.00	0.15	1	0.091	0.40	285.01
7	400.00	0.10	1	0.050	0.67	383.81	34	400.00	0.15	1	0.061	0.58	319.90
8	200.00	0.15	1	0.080	0.38	280.70	35	200.00	0.15	-1	0.050	0.34	222.40
9	300.00	0.10	1	0.064	0.66	348.49	36	300.00	0.10	-1	0.049	0.42	290.26
10	200.00	0.20	1	0.097	0.30	224.27	37	300.00	0.20	-1	0.052	0.30	237.05
11	400.00	0.20	-1	0.046	0.47	242.89	38	200.00	0.10	1	0.078	0.52	345.55
12	300.00	0.10	-1	0.048	0.43	291.04	39	300.00	0.15	-1	0.041	0.44	260.38
13	300.00	0.15	-1	0.053	0.41	261.82	40	300.00	0.10	1	0.073	0.64	351.78
14	400.00	0.10	-1	0.041	0.52	330.37	41	300.00	0.10	-1	0.049	0.44	288.00
15	200.00	0.15	-1	0.049	0.33	215.91	42	400.00	0.10	1	0.061	0.66	379.04
16	400.00	0.15	-1	0.044	0.49	288.87	43	200.00	0.20	-1	0.064	0.26	205.63
17	200.00	0.20	-1	0.058	0.25	200.28	44	300.00	0.15	1	0.072	0.51	292.54
18	300.00	0.15	1	0.074	0.52	280.48	45	400.00	0.20	1	0.064	0.55	225.29
19	400.00	0.20	-1	0.048	0.48	229.63	46	300.00	0.20	1	0.076	0.37	244.65
20	200.00	0.10	1	0.087	0.50	344.89	47	400.00	0.20	-1	0.030	0.46	233.31
21	400.00	0.10	-1	0.037	0.51	344.18	48	400.00	0.15	-1	0.038	0.49	285.02
22	300.00	0.15	-1	0.051	0.42	263.77	49	200.00	0.20	1	0.088	0.31	223.08
23	300.00	0.20	-1	0.050	0.32	231.41	50	400.00	0.15	1	0.060	0.54	314.86
24	400.00	0.10	1	0.058	0.68	384.66	51	200.00	0.15	-1	0.068	0.33	218.83
25	300.00	0.10	1	0.070	0.68	346.43	52	200.00	0.15	1	0.082	0.42	284.35
26	200.00	0.10	-1	0.048	0.40	292.39	53	200.00	0.10	-1	0.071	0.37	296.17
27	200.00	0.20	-1	0.056	0.29	202.39	54	400.00	0.10	-1	0.041	0.53	332.12

**Fig. 1:**
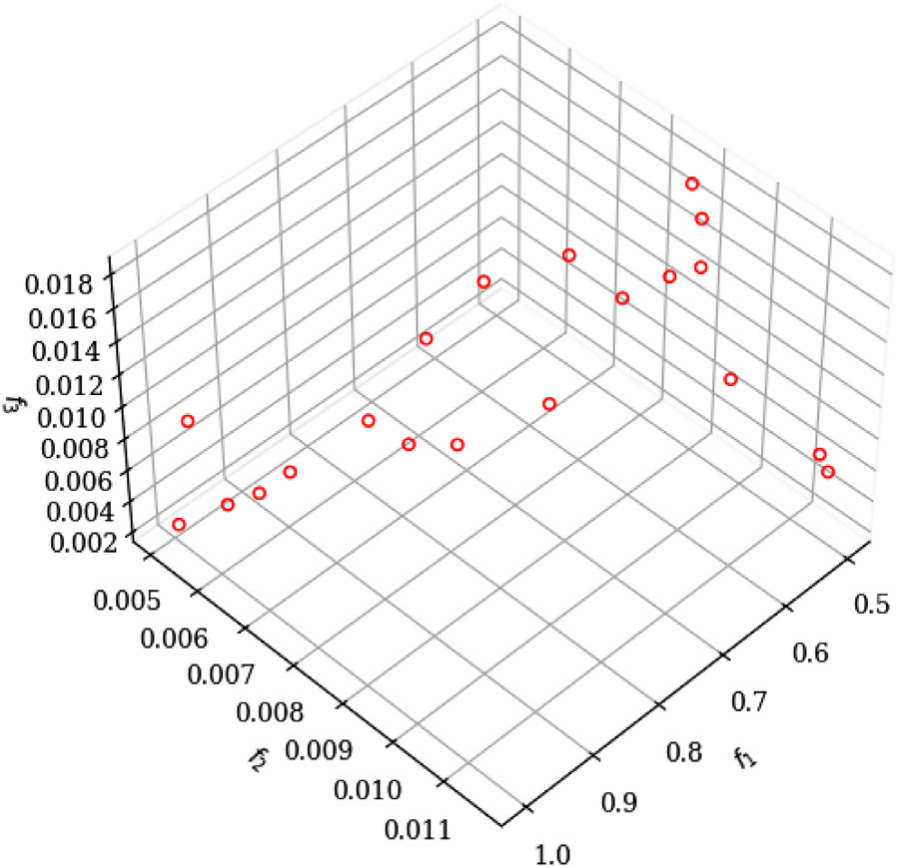
Graph obtained by executing the NSGA-II algorithm in python.

**Fig. 2:**
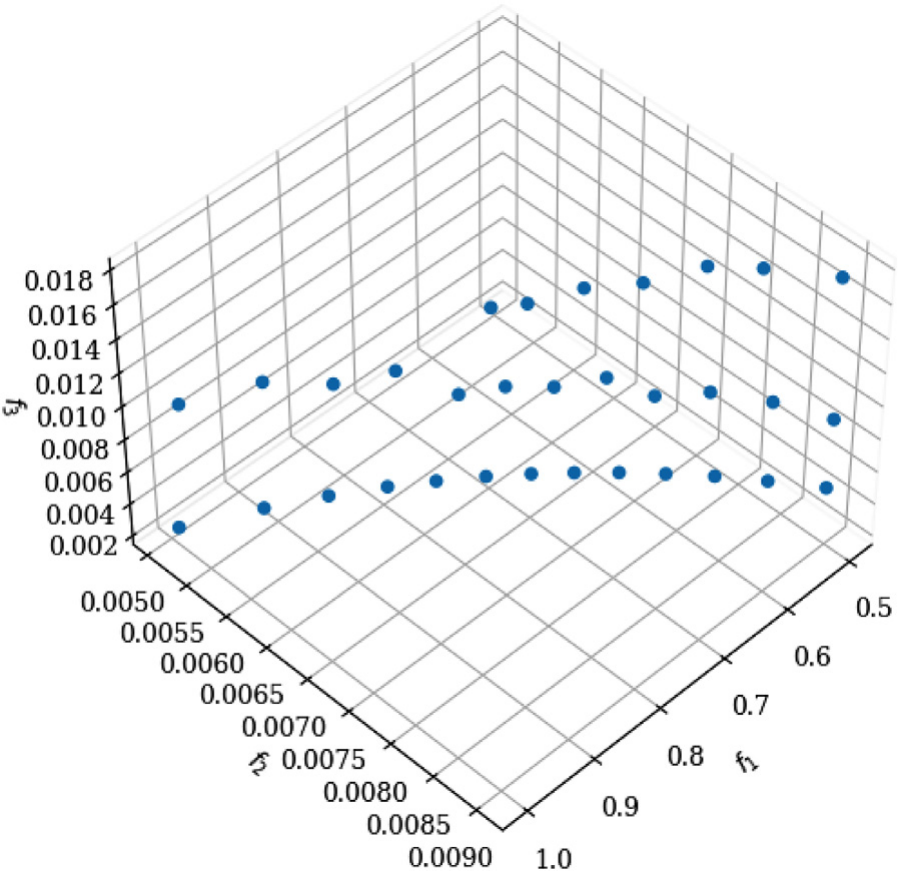
Graph obtained by executing the NSGA-III algorithm in python.

For the analysis, we started from a population of 54 variants shown in Table 1. The results of convergence of the Pareto front when arriving at the variants, in the NSGA-II and NSGA-III algorithms that are used, are presented in Tables 2 and 3 respectively. The optimal selection of values for the best approximation of the Pareto front is shown in Tables 4 and 5. The non-uniform distribution of the Pareto front points for the NSGA-II algorithm is presented in Fig. 1. In the case of the NSGA-III algorithm, it is shown in Fig. 2, where a better approximation between the points is observed in the graph. Its uniform distribution is due to the incorporation of reference points.

**Table 2:** The NSGA-II non-dominated classification algorithm shows in 20 variants, the results for the restrictions (t: machining time; E: energy consumption; MRR: Material Removal Rate).

Idx	f1=t	f2=VB	f3=E	MRR	V	f	a_p_
1	0.99993945	0.00510670	0.00250046	5.0010	100.00	0.10	0.50
2	0.50644165	0.00892512	0.01792375	35.847	100.00	0.20	1.81
3	0.50000000	0.01164106	0.00592223	11.844	118.00	0.20	0.50
4	0.50075722	0.00974574	0.00757691	15.154	105.00	0.20	0.72
5	0.50450523	0.01151685	0.00688382	13.768	118.00	0.20	0.59
6	0.67529866	0.00704592	0.01273727	25.475	100.00	0.15	1.72
7	0.98210653	0.00518261	0.00877409	17.548	100.00	0.10	1.72
8	0.73035554	0.00660768	0.00982330	19.647	100.00	0.14	1.43
9	0.61566775	0.00760087	0.00457847	9.1570	100.00	0.16	0.56
10	0.55596920	0.00826500	0.01100820	22.016	100.00	0.18	1.22
11	0.61166797	0.00790407	0.01439584	28.792	102.00	0.16	1.72
12	0.50000000	0.00905648	0.01595026	31.901	100.00	0.20	1.59
13	0.74955230	0.00646831	0.00334342	6.6870	100.00	0.13	0.50
14	0.90894281	0.00552236	0.00275088	5.5020	100.00	0.11	0.50
15	0.79009426	0.00619591	0.00544942	10.899	100.00	0.13	0.86
16	0.72142573	0.00709937	0.00391392	7.8280	104.00	0.14	0.54
17	0.94433719	0.00535265	0.00268540	5.3710	100.00	0.11	0.51
18	0.52006087	0.00872878	0.01232424	24.648	100.00	0.19	1.28
19	0.50208257	0.00910117	0.01317379	26.348	101.00	0.20	1.31
20	0.87901971	0.00576680	0.00379460	7.5890	101.00	0.11	0.66

**Table 3:** The NSGA-III non-dominated ordering algorithm shows the results for the restrictions presented in 32 variants.

Idx	f1=t	f2=VB	f3=E	MRR	V	f	a_p_
1	0.50088613	0.00900183	0.01755784	35.116	100	0.20	1.76
2	0.85760599	0.00579198	0.00291950	5.839	100	0.12	0.50
3	0.79628818	0.00615516	0.01021478	20.430	100	0.13	1.63
4	0.56417873	0.00816499	0.00444841	8.897	100	0.18	0.50
5	0.54224644	0.00843497	0.01724436	34.489	100	0.18	1.87
6	0.57420249	0.00804791	0.01690183	33.804	100	0.17	1.94
7	0.63589003	0.00740192	0.00977689	19.554	100	0.16	1.24
8	0.99999938	0.00510642	0.00252963	5.059	100	0.10	0.51
9	0.50010667	0.00901393	0.00924129	18.483	100	0.20	0.92
10	0.59480916	0.00781869	0.00420392	8.408	100	0.17	0.50
11	0.61335971	0.00762413	0.01554126	31.083	100	0.16	1.91
12	0.56915769	0.00810651	0.00955902	19.118	100	0.18	1.09
13	0.65730672	0.00720389	0.00380382	7.608	100	0.15	0.50
14	0.74523061	0.00649889	0.00858622	17.172	100	0.13	1.28
15	0.60377939	0.00772458	0.00892593	17.852	100	0.17	1.08
16	0.68898130	0.00693089	0.0036358	7.272	100	0.15	0.50
17	0.65259313	0.00724619	0.01499132	29.983	100	0.15	1.96
18	0.91762321	0.00548121	0.00273571	5.471	100	0.11	0.50
19	0.53290664	0.00855692	0.00956182	19.124	100	0.19	1.02
20	0.50141739	0.00899402	0.00499260	9.985	100	0.20	0.50
21	0.76434283	0.00636661	0.00327235	6.545	100	0.13	0.50
22	0.71972051	0.00668719	0.01373775	27.476	100	0.14	1.98
23	0.67270987	0.00706828	0.00905343	18.107	100	0.15	1.22
24	0.80550741	0.00609734	0.00311262	6.225	100	0.12	0.50
25	0.69253818	0.00690165	0.01397849	27.957	100	0.14	1.94
26	0.72393345	0.00665535	0.00346074	6.921	100	0.14	0.50
27	0.53312866	0.00855311	0.00468975	9.379	100	0.19	0.50
28	0.70879596	0.00677157	0.00902758	18.055	100	0.14	1.28
29	0.99198892	0.00514021	0.01004552	20.091	100	0.10	1.99
30	0.91293563	0.00550343	0.01047495	20.95	100	0.11	1.91
31	0.84978553	0.00583557	0.00976910	19.538	100	0.12	1.66
32	0.62580854	0.00749954	0.00399751	7.995	100	0.16	0.50

**Table 4:** Result of the best solutions for the NSGA-II algorithm.

SOLUTIONS	*OBJECTIVE*			VARIABLE	*CONTRAINS*		
Idx	f1=t	f2=VB	f3=E	MRR	V	f	ap
1	0.99993945	0.00510670	0.00250046	5.001	100.0	0.10	0.50
2	0.98210653	0.00518261	0.00877409	17.548	100.0	0.10	1.72
3	0.94433719	0.00535265	0.00268540	5.371	100.0	0.11	0.51
4	0.90894281	0.00552236	0.00275088	5.502	100.0	0.11	0.50

**Table 5:** Result of the best solutions for the NSGA-III algorithm.

SOLUTIONS	OBJECTIVE			VARIABLE	CONTRAINS		
Idx	f1=t	f2=VB	f3=E	MRR	V	f	ap
1	0.99999938	0.00510642	0.00252963	5.059	100.0	0.10	0.51
2	0.99198892	0.00514021	0.01004552	20.091	100.0	0.10	1.99
3	0.91762321	0.00548121	0.00273571	5.471	100.0	0.11	0.50
4	0.91293563	0.00550343	0.01047495	20.95	100.0	0.11	1.91

Fig. 1 shows the behavior for cutting speed V = 100 m/min, feed rate 0.1 < f < 0.2 mm/rev and cutting depth at 1.3 < ap < 1.8 mm. In this data set, the analysis of the objective functions showed that the energy consumption (0.013 ≤ E ≤ 0.018 kW. H/dm^3^) is minimized when performing the roughing operation, to reach the estimated production levels in a machining time *t* ≥ 0.5 mm; and initial tool wear behaves in the range of 0.007 ≤ VB ≤ 0.009 mm.

The constant cutting speed with Vc = 100 m/min, the feed rate behaves 0.1 < fn < 0.2 and the depth of cut at 0.5 < Ap < 1.2. In this state, in relation to the mentioned restrictions, the results of the studied objective functions showed fundamentally: it is achieved to decrease the energy consumption in the values of 0.015 ≤ E ≤ 0.017 for the material removal to reach the production levels in a time *t* ≥ 0.5, and with the tool wear in the values of 0.005 ≤ VB ≤ 0.009.

In the Table 4 show the analyzing the approximation with the pareto front, the behavior of the tool wear during the turning operation, the value of time (t) in the interval of (0.90–0.99) minutes per cubic decimeter has been taken as reference, in which the best behavior is appreciated, with the lowest and most stable values of feed rate (0.005) millimeters per minute. Meanwhile, the energy consumption is set at (0.002–0.08) kilo what hour per cubic decimeter, for a volume of material removed of 5–17 millimeters.

In Table 5, for the non-dominated sorting algorithm NSGA-III when implemented, with its approach to problems with three or more objective functions, the benchmarks are incorporated. In this technique, stacking distances between non-dominated solutions are substituted as the second selection criterion. These benchmarks represent the regions of the Pareto front that can be discovered, with the objective of maintaining the diversity of the population resulting from each generation. To discriminate these non-dominated solutions, the utility function, whose value indicates the relevance of a solution to approximate benchmarks, is used. When analyzing the behavior of tool wear when applying NSGA-III, during the turning operation, the four best behaviors have been taken, in relation to the time variable (t) in the interval of (0.91–0.99) minutes per cubic decimeter, in this range the best behavior of tool wear is observed, the lowest and most stable values of feed rate (0.005) millimeters per minute. A low energy consumption is achieved, set at (0.002–0.01) kilo what hour per cubic decimeter for a volume of material removed of (5–20 millimeters), all this reference of practical result with approximation to the Pareto front.

## 4 Experimental Design, Materials and Methods

### 4.1 NSGA-II and NSGA-III Algorithms

The authors propose a new method that uses the framework of the procedure based on the NSGA-II algorithm that works with a set of predefined reference points and demonstrates that they can solve problems optimization from 2 to 15 objectives.

### 4.2 Model Proposal and Description

The mathematical model for the optimization of the machining parameters analyzed in this work, is expressed for the following objectives:

$$mint=\frac{l}{f_{n}\cdot n}$$

$$minVB=\frac{V_{c}\cdot 1.52\cdot f_{n}\cdot 0.82}{3.25}*10000\;\left(Dry\right),minVB=\frac{V_{c}\cdot 1.42\cdot f_{n}\cdot 0.911}{2.33}*10000\;\left(MQL\right),$$

$$minE\frac{V_{c}\cdot a_{p}\cdot f_{n}\cdot k_{c}}{60}*10000.$$

Regarding the restrictions, the minimum and maximum values considered for the recommendations of the machining parameters (f, V_c_, a_p_) are provided by the manufacturers of the cutting tools in their catalogs. The previous objectives are subject to 0.10 < f < 0.20 (mm/r), 200 < V_c_ < 400 (m/min), 0.049 < a_p_ < 0.051 (mm). The multi-criteria optimization model has been developed to minimize the cutting tool wear and the energy consumption.

### 4.3 Statistical Analysis

In order to check the results of the variables analyzed in their approximation to the real values, with those obtained in the non-dominated evolutionary ordering methods, a statistical method was applied for their analysis. In Table 6, comparison of the objective functions with the NSGA-II and NSGA-III algorithms, with very close data to ensure the minimum values of time, energy and tool wear.

**Table 6:** Comparison of the results of the statistical analysis between the algorithms.

	NSGA-II				NSGA-III			
For Objective function	Min	Max	Mean	Standard deviation	Min	Max	Mean	Standard deviation
1	0.5	0.1	0.645	0.179	0.5	0.1	0.609	0.143
2	0.005	0.012	0.345	0.002	0.005	0.009	0.008	0.001
3	0.003	0.0018	0.007	0.005	0.003	0.018	0.005	0.009

## Ethics Statements

This work does not involve any type of human studies, animal studies, or data gathered using social media.

## CRediT authorship contribution statement

**Hiovanis Castillo Pantoja:** Conceptualization, Methodology, Software. **Alexis Cordovés García:** Conceptualization, Writing – original draft. **Roberto Pérez Rodríguez:** Writing – original draft, Software. **Ricardo del Risco Alfonso:** Software. **José Antonio Yarza Acuña:** Supervision, Visualization. **Ricardo Lorenzo Ávila Rondón:** Formal analysis, Supervision, Validation.

## Data Availability

NSGA-III (Reference data) (NSGA-III).
